# Deep learning-based seabird detection in fisheries for seabird protection

**DOI:** 10.1080/03036758.2025.2500998

**Published:** 2025-05-14

**Authors:** Jiawei Leong, Junhong Zhao, Bing Xue, William Gibson, Mengjie Zhang

**Affiliations:** a Victoria University of Wellington, Wellington, New Zealand; b Fisheries New Zealand, Ministry for Primary Industries, Wellington, New Zealand

**Keywords:** Seabird detection, object detection, YOLO, machine learning, deep learning

## Abstract

New Zealand is considered to be the ‘seabird capital’ of the world. As part of the harvesting process, some commercial fishers accidentally bycatch seabirds during fishing operations, which can result in accidental deaths and injuries. The accidental bycatch is impacting the long-term sustainability of New Zealand seabird populations. To address this, we developed a YOLO model that can be used to automatically detect seabirds that interact with the fishing vessels. The model development process involved gathering, annotating and preprocessing a new image dataset, conducting transfer learning across YOLO benchmark models, and performing hyperparameter tuning on the top YOLO models to further improve the model's performance. We evaluate the performance and effectiveness of our developed model under diverse data conditions, with it achieving a mAP@50 score of 0.9926 and a mAP@50-95 score of 0.9147 on the test data. The results demonstrate that the developed model performs effectively in unconstrained real-world marine scenarios, addressing the limitations of previous models primarily evaluated in controlled settings. This automation could help to reduce or even eliminate manual inspection of footages by reviewers and will help to quantify seabird interactions with commercial fishing vessels. Our contributions represent a significant first step in automated seabird detection, mitigating the gap between constrained and unconstrained real-world maritime scenarios.

## 1. Introduction

New Zealand (NZ)'s seabirds are considered to be a taonga (treasure) in Māori culture, with nearly one quarter of the world's seabirds breed in NZ (Te Ara The Encyclopedia of New Zealand 2015). With the vast ocean around NZ, its seafood is highly sought after around the world. However, the seafood exports have led to the bycatch of seabirds in fisheries. According to the summary data provided by Seafood NZ for the 12 Months ending in December 2023, a grand total of 233,552.7 tonnes of seafood were exported and earning a total of $2.11 Billion NZD in foreign exchange (Seafood New Zealand 2023). Different types of commercial fishing vessels such as trawls, set nets and longline vessels (Spark New Zealand Trading Limited 2024) are utilised to catch a wide variety of fish around NZ's Exclusive Economic Zone (EEZ) to sell to domestic consumers and/or exported for consumption overseas. One of the major threats to seabirds globally is the bycatch of seabirds in fisheries (Dias et al. 2019; Croxall et al. 2012) as during the seafood harvesting process, some commercial fishers accidentally bycatch seabirds which could result in deaths and injuries. Bycatch is defined as the incidental or unintentional capture of non-target species by the fishing gear (Ramírez et al. 2024; Hall 1996). This incidental bycatch is in some instances impacting the long-term sustainability of NZ seabird populations. The accidental deaths have a negative environmental impact as this is estimated to be causing some populations to decline (Edwards et al. 2023). According to Stats NZ, 90% of seabird species (or 86 of 96 seabird species) were classified either as ‘threatened with extinction’ or ‘at risk of becoming threatened’ as of December 2021 (Statistics New Zealand 2021). One of the innovative solutions to protect seabirds was proposed in a government cabinet paper. The proposal involves installing cameras onboard commercial fishing vessels to observe and capture footage of fishing operations (Fisheries New Zealand 2022). This ensures that fishers are complying with regulations put in place to protect seabirds and other protected species. This solution also provides a framework of site videos that can be further analysed. However, the video footages captured by the vessel's onboard camera systems are so vast in volume and duration that manual review by expert reviewers would be impractical and very time-consuming. To address this, we proposed a deep learning method to automate this process. This automation could help to reduce or even eliminate manual inspection of footages by expert reviewers and will help to quantify seabird interactions with the fishing vessels.

There were several methods proposed for detecting birds or seabirds in images and/or video clips. One example by Chen et al. (2023) developed eight different object detection models on a manually annotated dataset of birds in a wetland environment. Another example by Lei et al. (2023) developed a modified YOLOv7 detection model to detect small waterbirds also in a wetland environment using real-time video surveillance footages. However, these methods were primarily focussed on detecting land birds or wetland birds instead of seabirds in an ocean environment (i.e. onboard a fishing vessel) and the images or video footages were captured in a controlled environment of which the birds often appear as one close-up of the subject in the image instead of appearing in real marine interactions as small, moving birds in a group. Therefore, we propose our new method by constructing a new image dataset using video footages captured onboard fishing vessels and an object detection model that can be used to detect seabirds in the unconstrained marine scenarios.

The main objectives and contributions are as follows:

- Firstly, a new seabird detection dataset is created using video footages provided by Fisheries New Zealand (FNZ). This is achieved by analysing and drawing annotated bounding boxes for each seabird in each frame of the clip. After data annotation, the labelled dataset is then pre-processed so that it can be used by an object detection algorithm.
- Next, transfer learning is performed on ten different YOLO models for seabird detection: the nano, small, medium, large and extra-large YOLOv8 and YOLOv9 models. The ten models were evaluated to find the top two models that achieved the best performance, one model from YOLOv8 and the other from YOLOv9.
- After transfer learning, hyperparameter tuning was performed on the top two models using the grid search method to further improve the performance. Additionally, a basic interactive dashboard was constructed that can effectively visualise the detection results using the fine-tuned YOLO models.

## 2. Related work

### 2.1. Object detection models

Object detection is a technique in the field of computer vision that not only identifies objects (recognition/classification) but also locates objects of interest in the images (localisation). For our paper, the task is to classify whether an object is a seabird and also locate the seabird in an image. Two types of object detection models were considered for this paper: the Region-Based Convolutional Neural Network (R-CNN) and YOLO.

The R-CNN model features a large convolutional neural network that predict category independent region proposals for each object in an image (Girshick et al. 2014). The R-CNN also features a simple bounding box regression method that is used to predict the locations of where to draw a bounding box on the object of interest. One of the major limitations to the R-CNN model is that it is computationally intensive as the R-CNN will need to train three models separately. Firstly, a CNN to generate the image features, a classifier to predict the class and finally, a regressor to refine the bounding box coordinates (Bharati and Pramanik 2020). To overcome this limitation, Redmon et al. (2016) proposed a faster and more efficient object detection model titled the ‘You Only Look Once’ or the YOLO model. Unlike two-stage object detectors, YOLO is a one-stage object detector that features a single convolutional network that can simultaneously predict multiple bounding boxes and class probabilities for these bounding boxes. Instead of generating region proposals for each image, YOLO divides each image into a grid and predicts multiple bounding boxes and class probabilities for each grid at once. This enables YOLO to achieve faster inference speeds and greater accuracy than traditional R-CNNs models by giving it the ability to predict multiple bounding boxes per grid for an image. Therefore, this paper will utilise the YOLO object detection model for its advantageous speed and accuracy to detect objects in images.

**Figure 1.**
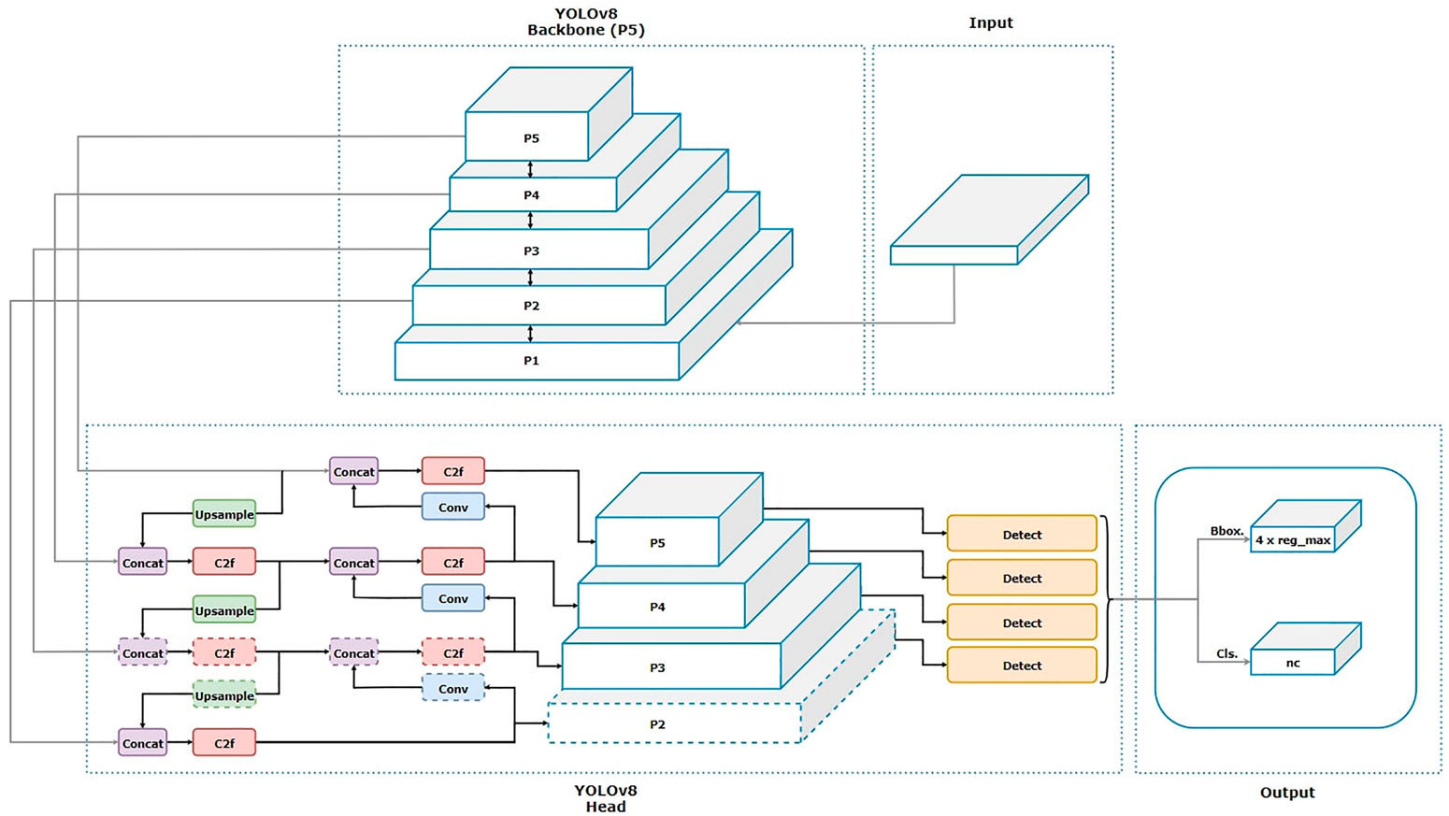
Simplified Backbone and head architecture diagram for YOLOv8 by Karna et al. (2023).

One of the more recent YOLO architectures is YOLOv8, which was developed by the Ultralytics team (Reis et al. 2023). The architecture for YOLOv8 is outlined in Figure 1. One of YOLOv8's key features is its anchor free split head which enables it to predict directly at the centre of an object instead of on the offset using anchor boxes (Torres 2024; Terven et al. 2023). This feature significantly reduces the number of bounding box predictions and speeds up model inference (Reis et al. 2023), which improves the model's accuracy for detecting seabirds of varying sizes and orientation. Another of YOLOv8's key features is its mosaic augmentation algorithm which augments the images during training. For every epoch, the model takes four random images from the training set, randomly crop each image and then stitches these into one image (Reis et al. 2023). Thus, improving the model's ability to generalise to new data such as detecting seabirds under various conditions and backgrounds. Another of the more recent YOLO architectures is YOLOv9, which was developed by Wang and Liao (2024). One of YOLOv9's key features is the Programmable Gradient Information (PGI) mechanism which generates reliable gradients through one or more auxiliary reversible branches without affecting the model's performance (Wang and Liao 2024; Yaseen 2024). The auxiliary reversible branch addresses the vanishing gradient problem by allowing the gradients to flow back without loss during backpropagation. Additionally, the use of reversible functions and the enhanced spatial attention layers ensures that the information is retained during training. These new features address the main issue of information bottlenecks, where information can be lost as data is transformed through a neural network, leading to more robust prediction and fewer detection errors (Potrimba 2024). For instance, this enables the detection of subtle features that can distinguish individual seabirds in complex object detection environments.

The YOLOv8 and YOLOv9 models were pre-trained on an existing dataset titled the Common Objects in Context (COCO) 2017 dataset, which is a collection of images from 80 different classes ranging from person, boat to apple and cake (Lin et al. 2015). However, these models cannot be applied directly to the current detection task of detecting seabirds in fish harvesting images or video frames as these YOLO models were trained for a multi-class task and has insufficient performance for seabird detection. Another reason is that our images are significantly different with the COCO dataset as the images in COCO 2017 dataset often have one object that occupy most of the image while ours contain seabirds and features seabirds from a vessel's onboard perspective. Additionally, the seabirds that were captured are often small in size and their appearance changes over time. Therefore, the detection tasks are different, and we will need to adapt the YOLO models to focus on detecting seabirds in real-world challenges.

### 2.2. Seabird detection methods

Several different object detection models such as the Faster R-CNN framework, and early versions of the YOLO model have been applied in practice to various real-world applications to detect birds or seabirds in images or video clips.

#### 2.2.1. Single images

Saxena et al. (2022) proposed a method to detect birds in images that were found on the internet. They utilised the Faster R-CNN model from the Detectron2 library (Wu et al. 2019) and the YOLOv5 object detection model to detect images of birds in the Oriental Bird Club Images Database (Cornell Lab of Ornithology n.d.), which contains 3000 images of birds. The authors found that the Faster R-CNN model performed poorly on the dataset with an Average Precision (AP) score of 0.27 whereas YOLOv5 scored a mAP score of 0.78 on the same dataset. Saxena et al. concluded that YOLOv5 is more efficient at detecting images of birds than the Faster R-CNN model.

Chen et al. (2023) tested eight different object detection models including YOLOv8 on a manually annotated bird dataset collected from images of ten different protected species of birds in a wetland environment. They found that the YOLOv7 model was the most efficient model for detecting whole birds in images with a mAP@50 of 0.932 across all bird classes. To further enhance the performance of the YOLOv7 model, they added three Global Attention Mechanism (GAM) modules on the head side of the model and constructed a new dataset containing 11,139 individual bird images for the multi-object tracking task. These changes improved the mAP@50 score to 0.951 and the mAP@50-95 score to 0.815. On the other hand, the YOLOv8 model managed to achieve a mAP@50 score of 0.927 on the test set across all bird classes for detecting whole birds in images. Additionally, this study explored utilising the DeepSORT algorithm to perform multi-object tracking in video clips.

#### 2.2.2. Frames from video footages

Hentati-Sundberg et al. (2023) employed the YOLOv5 model for seabird monitoring and research. They collected a total of 5600 hours of CCTV camera footage of seabirds on an island in Sweden and used YOLOv5 to generate the predictions on the annotated videos of adult seabirds, chicks and eggs. The YOLOv5 model produced the predictions for the location, size and the confidence score for all seabird detections frame-by-frame in each of the video clips. They found that the YOLOv5 model performed well with detecting the adult seabirds than detecting chicks and eggs with a precision and recall scores for the adult seabird class of 0.98 and 0.98 respectively. Furthermore, the authors added that their approach has the potential of utilising AI for real-time seabird detection (i.e. detecting seabirds as they appear in the camera feed) and they demonstrated how utilising the YOLOv5 object detection model can be used to accurately monitor breeding phenology for seabirds on an island.

Ma et al. (2024) utilised the YOLOv8 model to efficiently detect and monitor bird species on the Poyang Lake in China. They collected a total of 8077 high-quality video frames of birds from the surveillance cameras for five different species of birds. To overcome the object detection limitations of detecting high density objects and object occlusions, they proposed the YOLOv8-bird model that features a modified YOLOv8 nano model with various architectural modifications and a new loss function called the Inner-ShapeIoU loss function. The YOLOv8-bird model managed to achieve a mAP@50 and mAP@50-95 scores of 94.8% and 70.4% respectively. This work demonstrated that the proposed YOLOv8-bird model is well suited for bird detection and bird counting tasks for detecting and counting birds at Poyang Lake.

The existing work on detecting birds have focussed primarily on detecting close ups of a single or a flock of birds in an image, or detecting birds that are not in a marine environment (i.e. out at sea on a fishing vessel). Our paper will address these key limitations by developing a new seabird model specifically optimised for detecting individual or groups of seabirds in still frames from the video clips that were captured in challenging, unconstrained real-world maritime environments.

## 3. Datasets and data preprocessing

### 3.1. FNZ dataset

**Figure 2.**
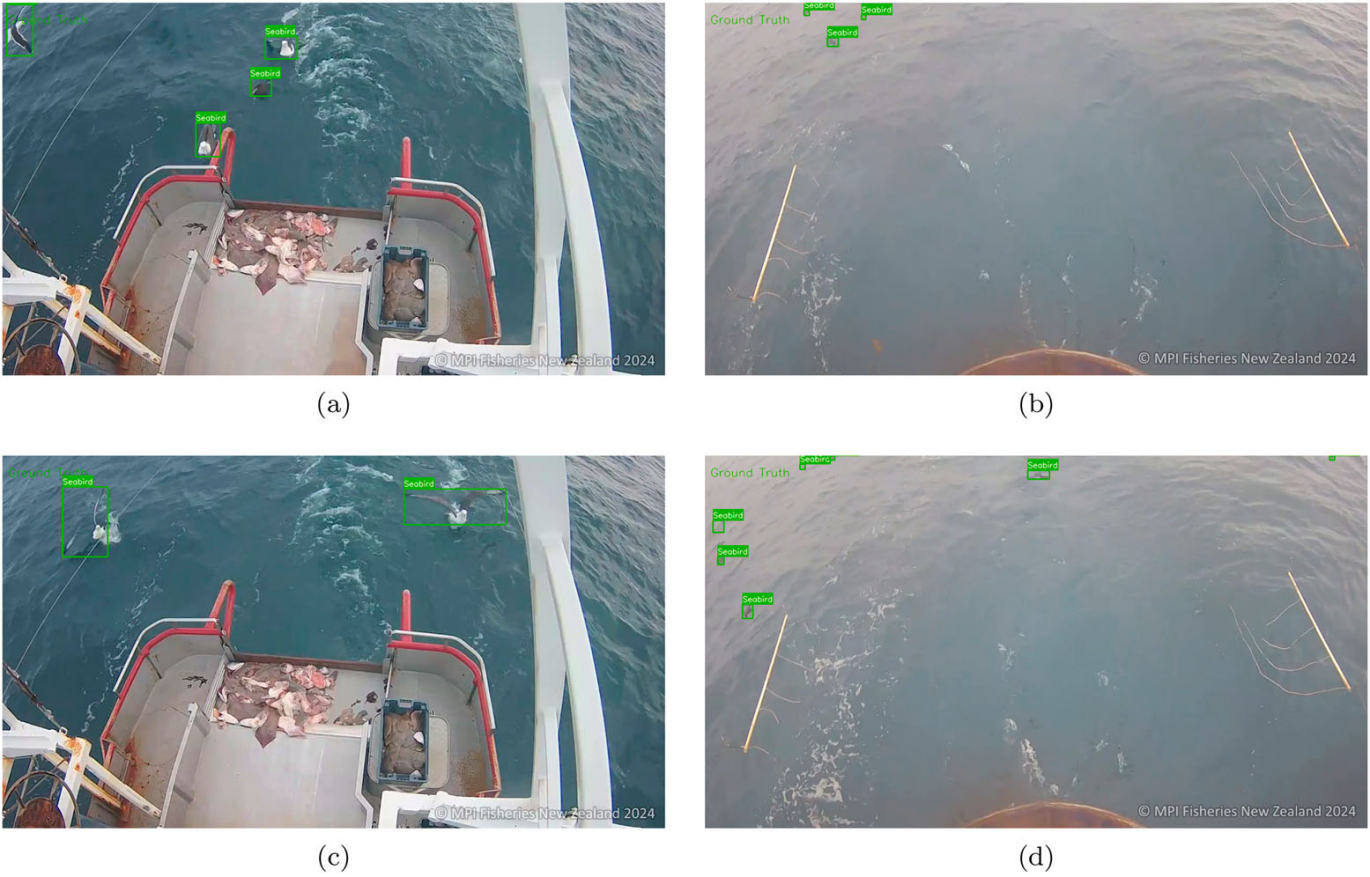
Example still frames from the onboard camera footages in the FNZ dataset with the ground truth bounding boxes.

The FNZ dataset is a pre-processed image dataset containing a total of 8402 annotated images from the video footages that were supplied by FNZ. This dataset consists of raw still images from the video footages that were captured on an onboard camera system installed on various commercial fishing vessels. Each image features individual seabirds or groups of seabirds within the frame of the camera with varying sizes. Some example images from this dataset with the ground truth bounding boxes are shown in Figure 2.

These raw footages do not come with any annotations for the seabirds and given that most object detection models typically require an annotated dataset to perform supervised learning on, every seabird will need to be annotated frame-by-frame before converting this into an image dataset. However, this process can often be labour and time intensive as it takes a significant amount of time and effort to find and draw bounding boxes for every bird in every frame in the videos.

For this paper, we annotated these footages by utilising a semi-automated approach. Firstly, a tracking algorithm is applied to create the initial tracked bounding boxes for each object, then we manually edit these bounding boxes to correct the position of each object. After data labelling has been completed, we pre-processed this dataset by extracting the sequence of frames from the videos to create an image dataset. Out of the four video clips that have been provided by FNZ, we selected two video footages to create the FNZ dataset. For privacy considerations, FNZ had carefully selected clips that contain no identity features in the videos (e.g. boat names, people, company logos, etc) before releasing these footages.

### 3.2. Data labelling

For this paper, we have employed the use of the Computer Vision Annotation Tool (CVAT) together with the built-in Transformer Tracking (TransT) algorithm (Chen et al. 2021) to label the images in the FNZ dataset. CVAT allows users to annotate images or videos using any of the four shapes: bounding boxes, 3D cuboids, Polygons, Polylines and key points. CVAT also allows interpolation of bounding boxes and automatic annotation using the TransT tracker to efficiently speed up the annotation process. This tool can be installed locally using Docker or can be accessed on the web. The TransT tracker can automate the labelling process without requiring a lot of manual effort to draw the bounding boxes and it can be integrated together with CVAT. The main disadvantage of CVAT is that the cloud version of this app has a quota limit on the number of TransT tracker calls and this requires a monthly subscription to continue using the TransT tracker.

## 4. Proposed method

### 4.1. Overall pipeline

**Figure 3.**
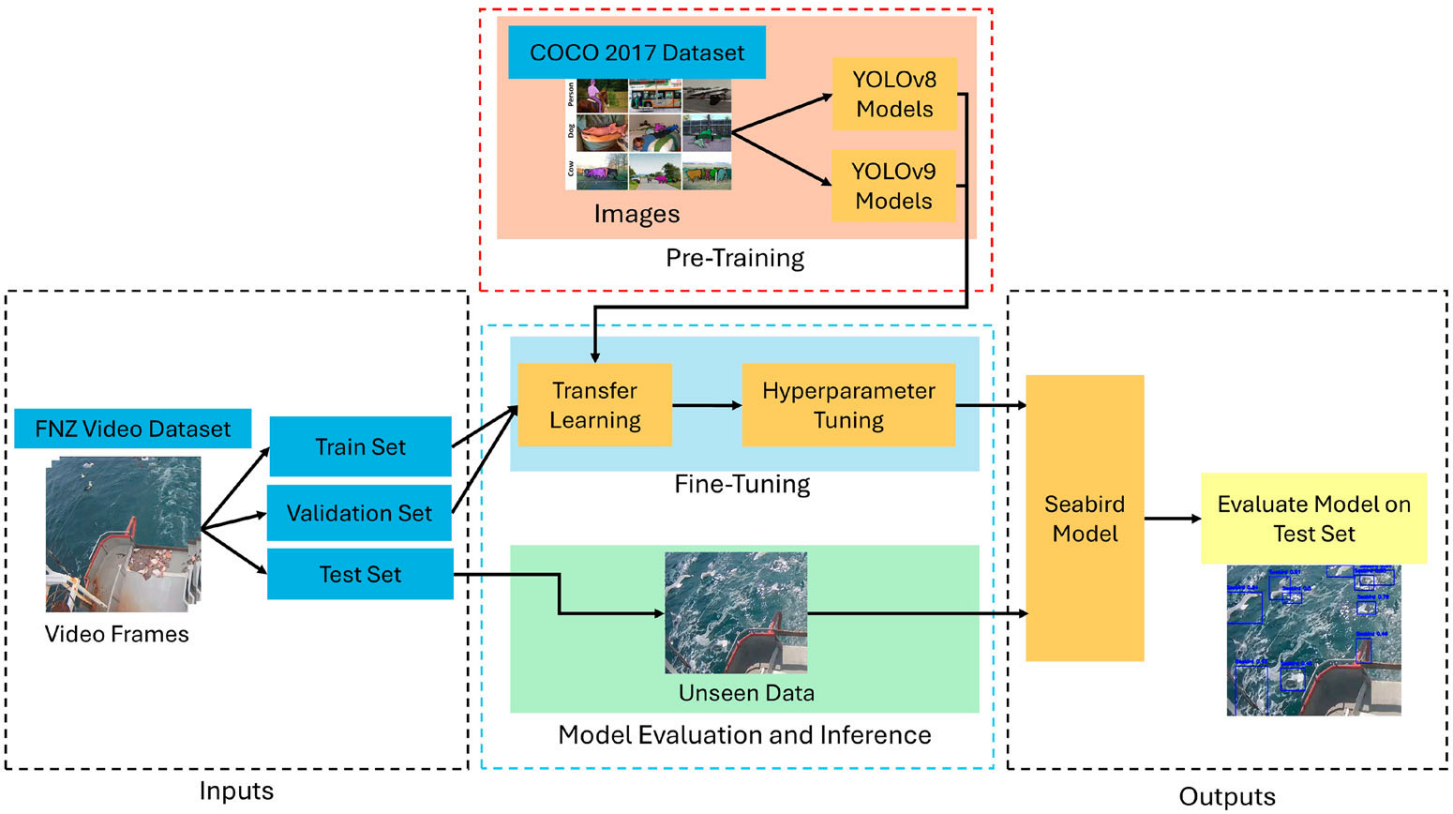
Model pre-training, fine-tuning and model evaluation/inference flowchart.

This paper focuses on fine-tuning various YOLOv8 and YOLOv9 models and finding an optimal set of hyperparameters to search for the model that achieves the best results on the test set. The YOLOv8 models were obtained through the Ultralytics Python package whereas the YOLOv9 models were obtained through the YOLOv9 GitHub repository by WongKinYiu.^1^ Figure 3 outlines the flowchart for the pre-training, fine-tuning and the model evaluation stages.

The first stage is to perform transfer learning on the FNZ dataset to tweak the models to this specific detection task for detecting seabirds only instead of detecting objects from the COCO 2017 dataset. Hyperparameter tuning was then performed on the best two models on the FNZ dataset to find the combination of hyperparameters that gives the best results on the validation set. The fine-tuned models from YOLOv8 and YOLOv9 are then compared with each other and the best model was selected based on the performance achieved on the validation set. This model was designated as the final seabird model. Finally, the seabird model can be evaluated on the test set in the model evaluation and inference stage. The seabird object detection model is an image object detection model which takes an image as input and outputs the detected objects in the image.

### 4.2. Pre-training stage

In the pre-training stage, all YOLO models shown in Table 1 had been trained on the COCO 2017 dataset containing 118K images in the training set and 5K images in the validation set with 80 object classes (Lin et al. n.d.). In total, there were ten models to fine-tune and evaluate on the FNZ dataset, ranging from the model with the smallest number of parameters to the model with the largest number of parameters. A YOLO model with fewer parameters is less complex but may struggle to capture some of the complex patterns in the dataset. In contrast, models with more parameters are more complex and has the ability to capture more complex patterns in the dataset. However, they are prone to over-fitting and may lead to poor results. Table 1 shows a comparison between the ten models on the COCO 2017 dataset.

**Table 1.** Model comparison for YOLOv8 & YOLOv9 models on the validation set of the COCO 2017 dataset.

	Model	No. Params (M)	No. Trainable Layers	FLOPs(G)	mAP@50-95 (%)	mAP@50 (%)
YOLOv8	Nano	3.2	225	8.7	37.3	52.6
	Small	11.2	225	28.6	44.9	61.8
	Medium	25.9	295	78.9	50.2	67.2
	Large	43.7	365	165.2	52.9	69.8
	Extra Large	68.2	365	257.8	53.9	71.0
YOLOv9	Tiny${}^{a}$	2.0	917	7.7	53.1	38.3
	Small	7.2	917	26.7	46.8	63.4
	Medium	20.1	603	76.8	51.4	68.1
	Compact	25.5	618	102.8	53.0	70.2
	Extended	58.1	1225	192.5	55.6	72.8

Note: Ultralytics (2023b), Wang and Liao (2024), Wong (2024), Ultralytics (2023c), and Ultralytics (2024)

${}^{a}$The tiny YOLOv9 model was released after the publication of the YOLOv9 paper

### 4.3. Fine-tuning stage

#### 4.3.1. Transfer learning

To adapt the YOLO models for seabird detection, we modified the detection head to output a single class for both architectures. As shown in Table 2, the pre-trained YOLO models were configured to detect classes for the COCO dataset. Our fine-tuned models were modified to detect seabirds only by changing the number of classes parameter (*nc*) from detecting eighty classes in the COCO dataset to a single class only and updating the model's weights based on FNZ's dataset. This modification ensures that our fine-tuned models focus solely on detecting seabirds only exclusively for FNZ's dataset. The rest of the YOLO architectures were not modified.

**Table 2.** Summary of changes made to the pre-trained YOLOv8 and YOLOv9 models.

Model	Type	No. Output Classes (nc)	Trained/Fine-Tuned On
YOLOv8 Large	Fine-tuned	1	FNZ Dataset
	Pre-trained	80	COCO 2017 Dataset
YOLOv9 Medium	Fine-tuned	1	FNZ Dataset
	Pre-trained	80	COCO 2017 Dataset

In the fine-tuning stage, transfer learning was performed first on ten different YOLO models. The optimal number of epochs was obtained by performing early stopping using the *patience* hyperparameter of the YOLO model. This parameter aims to stop model training if there have been no improvements of performance on the validation set for pre-defined number of epochs (e.g. 10 epochs) (Ultralytics 2023a). The purpose of transfer learning is to leverage the weights learned by the pre-trained YOLO models on the COCO dataset and adapt it to the current task of detecting seabirds. Each model was trained on the FNZ training dataset with the default hyperparameters for a preset maximum of 800 epochs, but training can be stopped early if the performance on the validation set plateaus. Once all ten models were evaluated, we will select the top two models that achieved the best performance to increase the default image resolutions for these models. The image resolution ensures that all input images were resized to a set image resolution in order to maintain the same resolution during training (Ultralytics 2023a). The image resolution hyperparameter was increased from its default resolution of 640 x 640 pixels to 736 x 736 pixels in order to capture the finer details in the input images, particularly for seabirds that appear as small objects in the images. However, the image resolution was still constrained by the available GPU memory.

#### 4.3.2. Hyperparameter tuning

After the transfer learning process, a greedy hyperparameter search was conducted by tuning a set of hyperparameters to search for the best model. To reduce the computational time and resources when tuning the models, we focussed on the two key hyperparameters of the model: the *learning rate* and *momentum*. Three different tuning methods were proposed to tune the YOLO models: manual tuning, grid search and a genetic algorithm that is built into YOLOv8 and YOLOv9. The grid search algorithm was selected instead of the built-in genetic algorithm because, unlike the genetic algorithm which tunes all the hyperparameters at once that could waste computational resources and increase the training time, grid search tunes a specific set of hyperparameters in a grid-like search space. Furthermore, manually tuning the hyperparameters was not used as it would take too much time to manually tune each of the model's hyperparameters. Thus, grid search offers a good balance between thoroughness and computational cost when tuning a focussed set of hyperparameters. We employed the grid search hyperparameter tuning method to systematically explore the learning rate (between 0.001 and 0.0001) and momentum values (between 0.9 and 0.95) in a smaller hyperparameter search space.

## 5. Experimental setup

### 5.1. Experiment overview

Both the quantitative results of the models (i.e. results from the performance metrics) and the qualitative results (e.g. analysing the detection results from the test set) of the models are analysed. For all runs of the YOLO model, the seed has been set to 0 to ensure the results are reproducible. All experiments were executed on a single RTX 6000 Graphics Processing Unit (GPU) device. The list of hyperparameters used to fine-tune the models are outlined in Table 3 with the hyperparameters to be used in the grid search tuning process highlighted in bold.

**Table 3.** Training Hyperparameters used.

Hyperparameter	Value
Epochs (initial)	800
Batch size	64
Image Size (initial)	640×640
Image Size (later)	736×736
**Initial Learning Rate**	0.01
Final Learning Rate	0.01
Optimizer	SGD
**Momentum**	0.937

For the FNZ dataset, all frames extracted from the two video footages were randomly shuffled and split into training, validation and test set with an 80%–10%–10% distribution. This resulted in 6721 images for the training set, 840 images for the validation set and 841 images for the test set for the FNZ dataset.

### 5.2. Performance metrics

Performance metrics are crucial for measuring the performance of object detection machine learning models. The performance metrics to be used in this paper are outlined below:

- Average Precision (AP) measures model's precision and recall performance by calculating the area under the precision-recall curve (Everingham et al. 2010).
- The *Mean Average Precision (mAP)* averages the Average Precision (AP) scores over multiple classes to evaluate the model's overall performance (Padilla and Netto 2020).
- The *Intersection Over Union (IoU)* measures the overlap between the predicted bounding box and the ground truth bounding box (Everingham et al. 2010).
- The *mAP@50 score* calculates the mAP at a fixed IoU threshold of 0.5 (Everingham et al. 2010), which means the model is evaluated based on its ability to detect objects with at least 50% overlap between the predicted and the ground truth bounding boxes. This score measures the model's effectiveness on simpler detection tasks.
- The *mAP@50-95 (or mAP) score* calculates the mAP score across ten IoU thresholds from 0.5 to 0.95 with a step size of 0.05 (Ren et al. 2016). The final score was calculated by averaging all the mAP scores across multiple IoU thresholds. This score measures how well the model performs across a range of detection tasks, from easier to more challenging ones such as detecting smaller objects.

For this task, the mAP score was chosen over the F1 score as mAP score considers multiple IoU thresholds (e.g. 50%, 75% and 95%) which can provide useful insights into the model's object detection performance. Whereas the F1 score only provides a specific trade-off (or the harmonic mean) between precision and recall at a fixed confidence threshold, it does not consider the IoU threshold. Additionally, the mAP score focuses on both classification and localisation accuracy due to the use of the IoU thresholds whereas the F1 score only considers the classification accuracy.

## 6. Results and discussion

This section presents the results of performing transfer learning, grid search tuning and the model comparison between the fine-tuned YOLOv8 model and the fine-tuned YOLOv9 model. The number of epochs, precision, recall, mAP@50 and the mAP@50-95 scores were evaluated. In addition to the results, the training time were recorded as days, hours, minutes and seconds (D:H:M:S).

### 6.1. Transfer learning results

We first fine-tuned each YOLO model with the default hyperparameters for a maximum of 800 epochs to ensure that the models converge at an optimal (or close to the optimal) number of epochs and the bias-variance trade-off has been stabilised. For this experiment, the patience hyperparameter was set to 30 epochs. Table 4 shows the early stopping results for all ten YOLO models for 800 epochs on the validation set for the FNZ dataset.

**Table 4.** Early stopping results for the YOLOv8 and YOLOv9 models on the validation set.

	Model	Best Epochs	Precision	Recall	mAP@50	mAP@50-95	Training time
YOLOv8	Nano	395	0.9539	0.9027	0.9614	0.6676	00:08:28:55
	Small	435	0.9484	0.9257	0.9721	0.7136	**00:06:41:29**
	Medium	608	0.9482	0.9303	0.9704	0.7334	00:15:47:32
	**Large**	519	0.9485	**0.9355**	**0.9751**	0.7414	00:19:37:49
	Extra Large	730	**0.9632**	0.9246	0.9738	**0.7502**	01:16:46:56
YOLOv9	Tiny	736	0.9491	0.8985	0.9544	0.6443	01:13:40:23
	Small	595	0.9469	0.9224	0.9697	0.6963	**01:03:26:58**
	Medium	579	**0.9618**	0.9124	0.9647	0.7028	01:11:52:45
	**Compact**	754	0.9407	**0.9256**	**0.9698**	**0.7305**	02:10:03:23
	Extended	697	0.9530	0.9160	0.9646	0.7113	03:07:42:10

Of all the YOLOv8 models tested, the large model managed to score the highest mAP@50 score on the validation set. However, the extra-large model achieved a marginally better mAP@50-95 score than the large model. The decision was made to select the large model as the extra-large model was more computationally expensive and required more time to train than the large model.

Of all the YOLOv9 models tested, the compact model achieved the best mAP@50 score on the validation set while the extended model took the longest time with three days and seven hours to train, making it the most computationally expensive model to train. The decision was made to select the medium model instead of the compact model as the medium model provided a good trade-off between the accuracy and the computational complexity (e.g. the model's size and the training time). This is because larger and more complex models may lead to longer training and inference times which will not be suitable for a fast, real-time monitoring application. While this sacrificed a bit of the improved performance, this decision cuts back the training time required to train the YOLOv9 model.

In Table 4, we noticed that some of the smaller YOLO models (e.g. YOLOv9 tiny and small) took more epochs and time to train than the rest of the YOLO models. For example, YOLOv9 small took 595 epochs to train whereas YOLOv9 medium took 579 epochs to train. One explanation for these intriguing observations could be that during model training, these models exhibited unstable validation metrics (i.e. the fluctuation of the mAP@50 and/or the mAP@50-95 scores) which affected the early stopping mechanism for these models and leading to slower convergence.

After the top two models were selected, we then increase the default image resolutions for the YOLOv8 and YOLOv9 models. As shown in Table 5, we can see a significant improvement in results when the image resolution was increased from 640×640 pixels to 736×736 pixels on the test set. However, we observed a longer training time with an increased image resolution. From the table, the YOLOv8 large model managed to achieve a better mAP@50 score than the YOLOv9 medium model.

**Table 5.** Image resolution results for the selected models on the test set.

	Model	Epochs	Image Res.	Precision	Recall	mAP@50	mAP@50-95	Training time
YOLOv8	Large	519	640	0.9737	0.9651	0.9891	0.8926	00:19:37:49
YOLOv8	Large	519	736	0.9868	0.9713	**0.9932**	**0.9165**	01:00:49:19
YOLOv9	Medium	579	640	0.9413	0.9215	0.965	0.7142	01:11:52:45
YOLOv9	Medium	579	736	0.951	0.936	**0.9708**	**0.7149**	01:20:40:31

### 6.2. Grid search tuning results

The results after performing grid search tuning on the validation set for the FNZ dataset for the YOLOv8 large and the YOLOv9 medium models are shown in Table 6. The YOLOv8 large model that was fine-tuned for 519 epochs with a learning rate of 0.01 and a momentum of 0.95 managed to achieve the best mAP@50 score on the validation set out of the three other models tested. Therefore, we picked this specific combination of hyperparameters for the YOLOv8 model for the final comparison. Out of the four YOLOv9 medium models that were tested, the model that was fine-tuned for 579 epochs with a learning rate of 0.01 and a momentum of 0.95 managed to achieve the best mAP@50 score on the validation set. Similar to the YOLOv8 model, we picked this specific combination of hyperparameters for the YOLOv9 model for the final comparison.

**Table 6.** Grid search results for finding the optimal model on the validation set.

	Model	Learning Rate	Momentum	Precision	Recall	mAP@50	mAP@50-95
YOLOv8	Large	0.01	0.9	0.95403	0.9465	0.97404	0.75147
	**Large**	**0.01**	**0.95**	0.96606	0.94418	**0.97495**	**0.75457**
	Large	0.001	0.9	0.95592	0.93981	0.97444	0.74185
	Large	0.001	0.95	0.94956	0.95131	0.97279	0.74488
YOLOv9	Medium	0.01	0.9	0.95168	0.94061	0.97412	**0.70717**
	**Medium**	**0.01**	**0.95**	0.94973	0.94596	**0.97545**	0.70692
	Medium	0.001	0.9	0.9495	0.92991	0.97196	0.69848
	Medium	0.001	0.95	0.95331	0.93945	0.96954	0.70327

**Figure 4.**
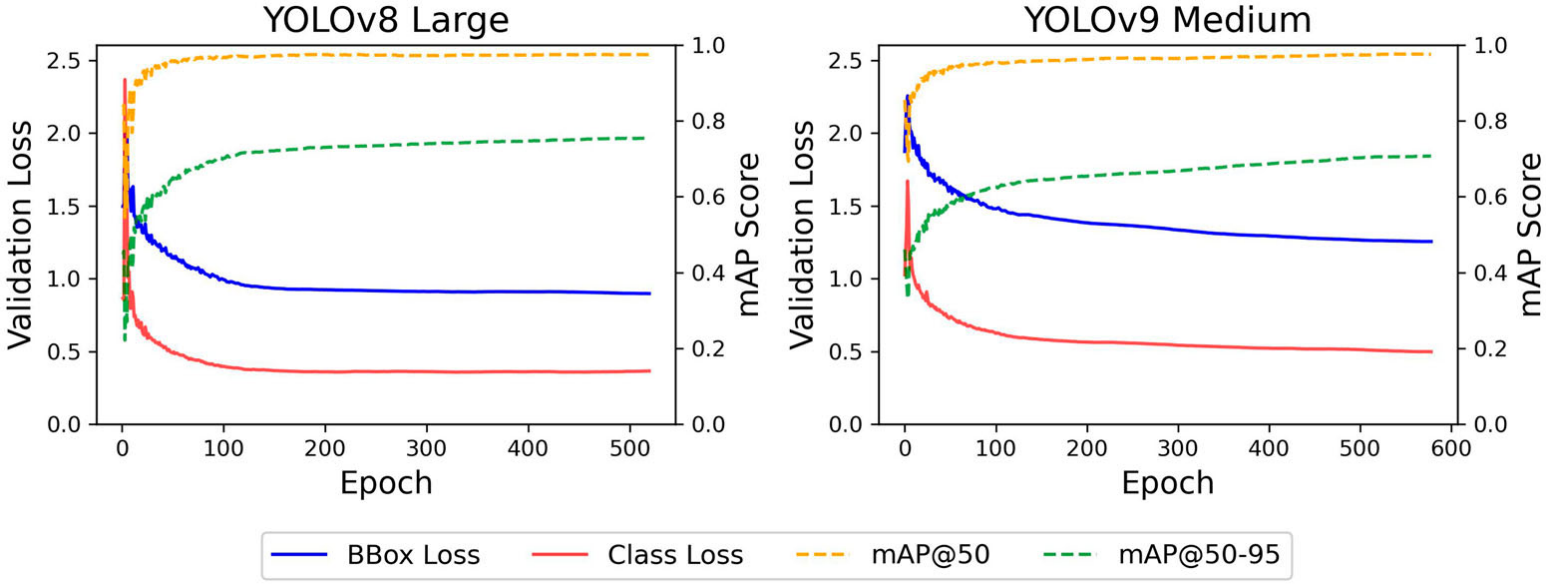
Validation losses and mAP plots for the fine-tuned YOLOv8 large and YOLOv9 medium models.

Using the grid search selected models, a final comparison on the test set was made to determine the best model for detecting seabirds. The test results on the FNZ dataset are presented in Table 8 with the YOLOv8 large model achieving significantly better mAP@50 and mAP@50-95 scores on the test set than for the YOLOv9 medium model of 0.9926 and 0.9147 respectively. In addition to the training time, the CPU inference time for the model to run detection on a single image of seabirds was recorded as seconds (s). Finally, Figure 4 visualises the validation losses and mAP metric plots for the fine-tuned YOLOv8 large and YOLOv9 medium models.

**Table 8.** Comparison between the YOLOv8 large model with and without fine-tuning on the test set.

	Model	Epochs	Precision	Recall	mAP@50	mAP@50-95	Training time	CPU Inference Time
**YOLOv8**	**Large**	519	0.9857	0.9723	**0.9926**	**0.9147**	**01:00:48:24**	4 s
YOLOv9	Medium	579	0.9815	0.9691	0.9917	0.8349	01:16:53:48	5 s

As illustrated in Figure 4, the fine-tuned YOLOv8 large model managed to converge faster than the fine-tuned YOLOv9 medium model. For the fine-tuned YOLOv8 large model, the bounding box and class validation losses plateaued after 100 epochs and took only a day to train, whereas for the fine-tuned YOLOv9 medium model, the validation losses plateaued after 500 epochs and took one day and sixteen hours to train. The fine-tuned YOLOv8 large model converged faster because its architecture prioritised speed by using an anchor-free detection head and the C2f module, whereas the architecture for YOLOv9 prioritised minimising information loss (Wang and Liao 2024), which increases the computational complexity for the YOLOv9 models.

### 6.3. Quantitative and qualitative comparisons

**Figure 5.**
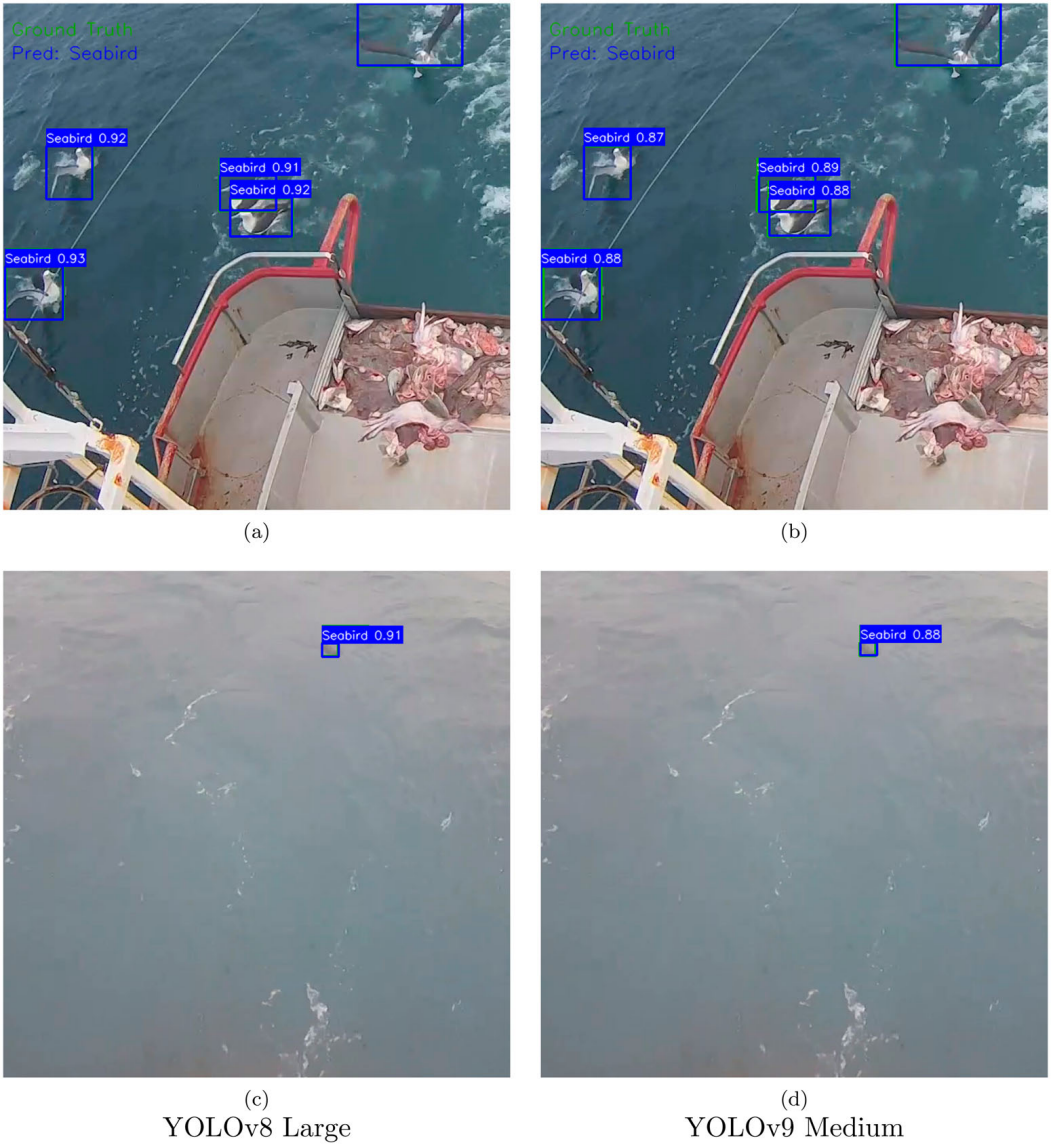
Ground truth and model predictions on images in the test set for the top two models.

Based on the experimental results, the fine-tuned YOLOv8 large model achieved superior performance across all metrics with a mAP@50 score of 0.9926 on the test set for the FNZ dataset, exceeding our mAP@50 target threshold of 90%. In other words, 99.26% of the time, the model's predicted bounding boxes overlapped with the ground truth bounding boxes by at least 50%. This exceptional score suggests that our model excels at handling the basic seabird detection tasks, particularly when identifying seabirds that are closer to the camera. The fine-tuned YOLOv8 large model demonstrated significant performance improvements over the YOLOv8 large model without any fine-tuning performed on this model as shown in Table 7. While there was only a modest increase in the mAP@50 score when compared to the pre-fine-tuned YOLOv8 large model from 0.9769 to 0.9926, we observed a significant improvement of the mAP@50-95 score from 0.7917 to 0.9147. In other words, 91.47% of the time, the model's predicted bounding boxes overlapped with the ground truth bounding boxes by at least varying IoU thresholds between 50% to 95%. This enhancement indicates that our fine-tuning process created a more robust YOLOv8 model capable of detecting seabirds at varying degrees of detection difficulty on the precision-recall curve such as detecting individual seabirds in flocks and distant seabirds that are further away from the camera. The model showed particular strength in detecting both the closer and distant seabirds within the same image with strong confident scores as shown in Figures 5A,C. We also observed the higher confidence scores for the YOLOv8 large predictions than for the YOLOv9 medium predictions on the same images, which indicates that the YOLOv8 large model is more confident that the objects detected were seabirds.

**Table 7.** Grid search selected model final comparison on the test set.

	Epochs	Learning Rate	Momentum	Precision	Recall	mAP@50	mAP@50-95	Training time
Fine-tuned	519	0.01	0.95	0.9857	0.9723	0.9926	0.9147	01:00:48:24
Without fine-tuning	100	0.01	0.937	0.9726	0.9224	0.9769	0.7917	00:03:42:30

**Figure 6.**
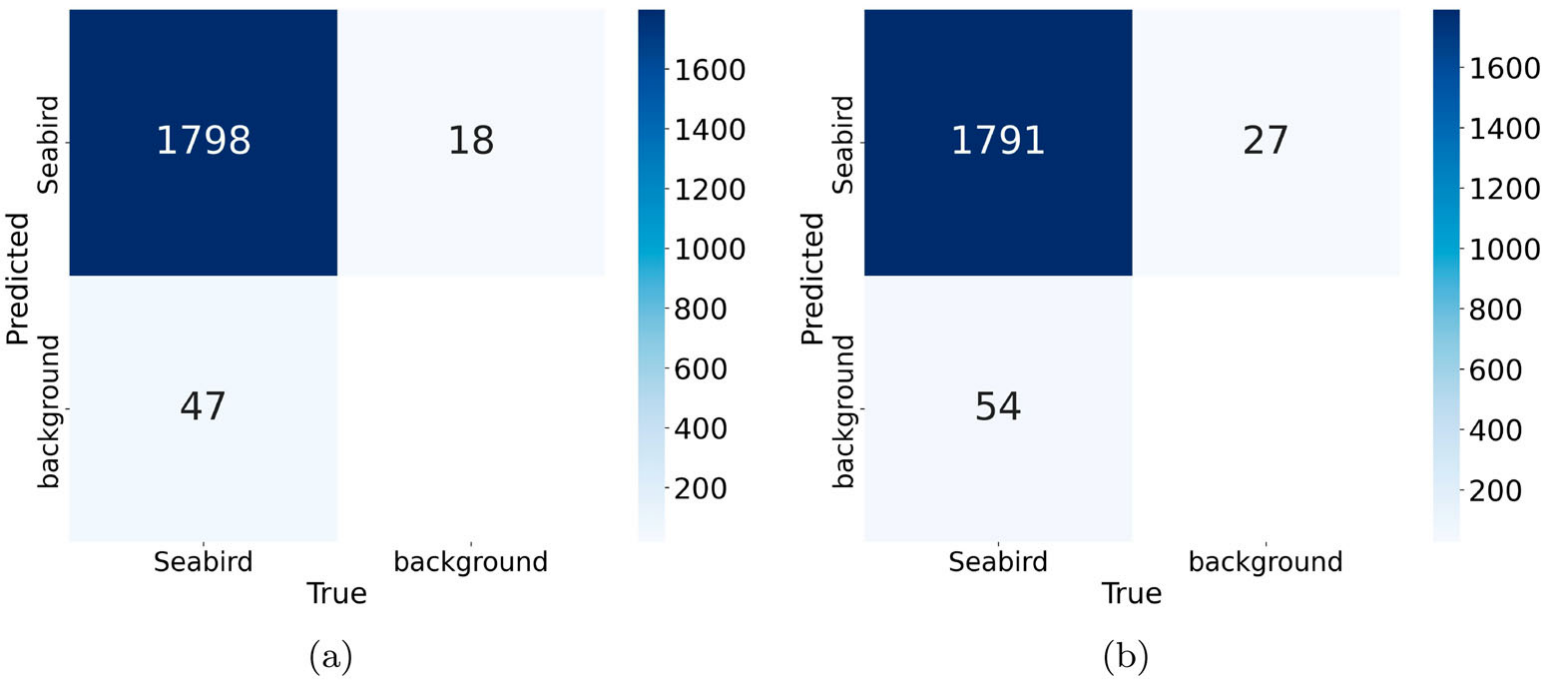
Confusion matrices for the FNZ dataset. **A**, Fine-tuned YOLOv8 large model. and **B**, Fine-tuned YOLOv9 medium model.

While the fine-tuned YOLOv9 medium model showed similar mAP@50 scores on the test set when compared to the YOLOv8 model, the significantly higher mAP@50-95 score from our fine-tuned YOLOv8 large model indicates superior performance in the more challenging detection scenarios than the fine-tuned YOLOv9 model. The confusion matrices provide additional support for our model's effectiveness which shows that the fine-tuned YOLOv8 large model in Figure 6A produced fewer false positives and false negatives when compared to the fine-tuned YOLOv9 medium model in Figure 6B. However, we observed that the fine-tuned YOLOv8 large model tends to produce more false negatives than false positives which indicates that the model is missing out on more potential seabird detections than detecting objects that are not seabirds. Additionally, as shown in Table 8, the CPU inference time for this model to run on a single seabird image is marginally shorter by one second than for the fine-tuned YOLOv9 model. This indicates that the YOLOv8 model is a more efficient YOLO model for real-time seabird detection.

After comparing the two models, we renamed our best model which is the fine-tuned YOLOv8 large model to the seabird model.

### 6.4. Dashboard development for real world use

**Figure 7.**
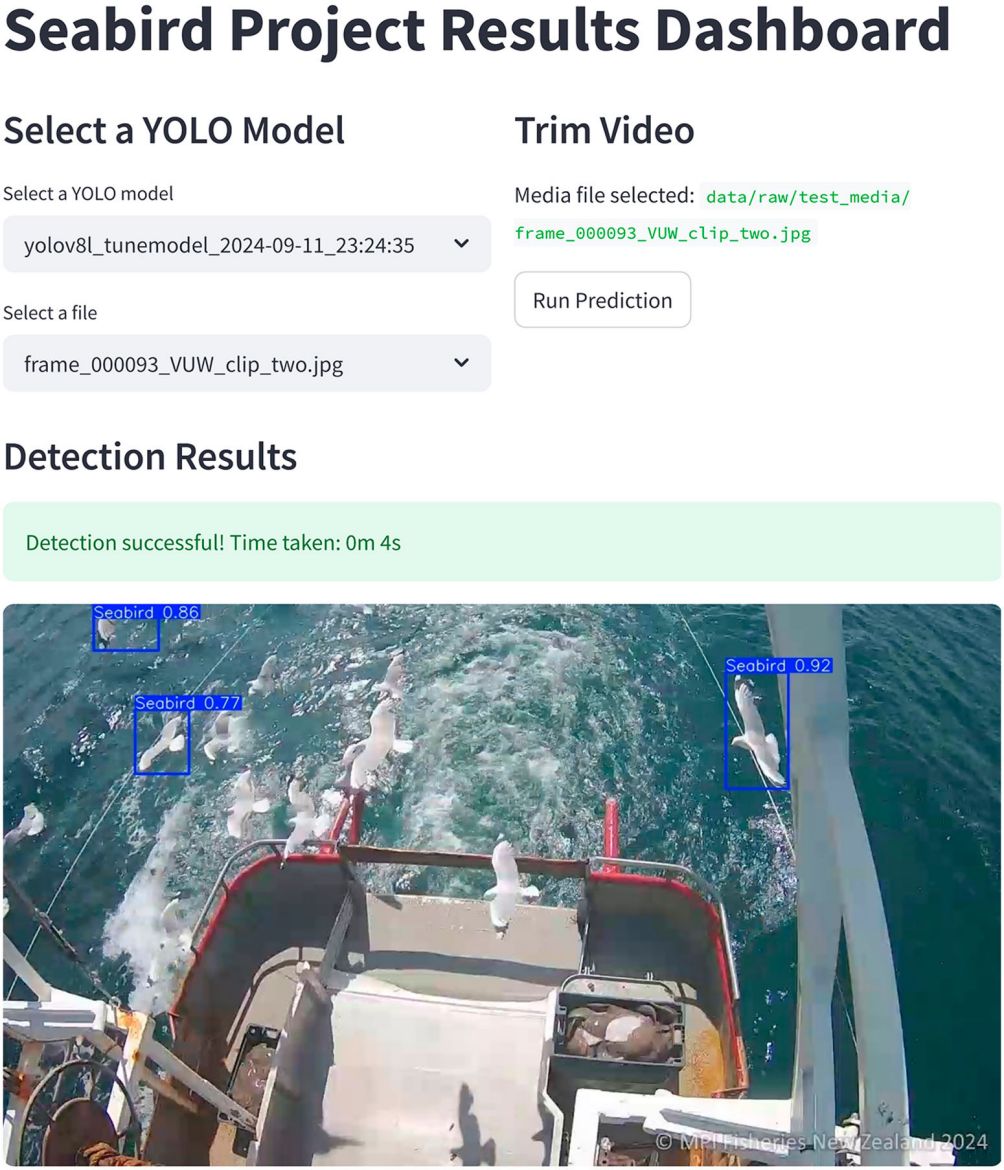
The interactive dashboard to visualise the detection results from the YOLO models.

To see the detection results on a still image, we constructed a basic interactive dashboard for this project to facilitate real-world usage as shown in Figure 7. The user can select a model from the dropdown menu and an image that they would like to run detections on. The model will run inference on this image and the result as shown here will be the inference results from the model.

This software provides a proof-of-concept for automated seabird monitoring in maritime environments, representing a significant first step forward towards the automated detection of seabird bycatch during fish harvesting operations on commercial fishing vessels. The seabird model can be extended to track the number of seabirds detected and how many were accidentally caught with the potential for identifying different species, such albatrosses, to determine which species are the most affected. Additionally, this automated monitoring approach enables fishers to better understand seabird interactions with commercial fishing vessels while supporting regulatory compliance and conservation efforts. For instance, this automated software could help to inform evidence-based policy making for seabird conservation and protection in New Zealand. Thus, helping fishers maintain sustainable fishing practices without requiring additional labour effort. Finally, this technology has broader applications beyond New Zealand waters and has the potential to be deployed on any fishing vessels worldwide.

### 6.5. Limitations

**Figure 8.**
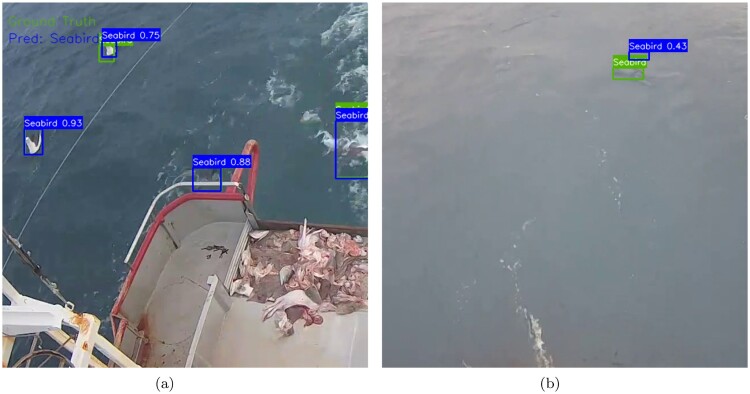
Detection results for the seabird model. **A** Detection with lower confidence and **B** Missed detection.

One of the limitations of our seabird model is the model's uncertainty in detecting some smaller seabirds that are further away from the camera under poor lighting conditions. For example, Figure 8A shows a detected seabird with a lower confidence score of 0.75 than the rest of the detections of 0.88 or above, suggesting the model's uncertainty in some cases. Additionally, in Figure 8B, the model missed one of the seabirds with a significantly lower confidence score of 0.43 which is considered to be a false negative. Some potential solutions to address these limitations include improving the backbone of the deep network architecture, tracking the motions of seabirds with varying sizes, and building a pre-processing and augmentation pipeline to enhance the input images for improved detection performance. Finally, our seabird model is only focussed on detecting a single class (‘Seabird’) and it cannot be used to detect certain species of seabirds (e.g. seagull, albatross or a shag). Automatic monitoring systems often require systems to be able to distinguish different species of seabirds. Thus, detecting seabirds can serve as the first step in locating the birds, which can then be used as a foundation for future work on identifying specific species of seabirds.

In addition to the limitations of our seabird model, our current FNZ dataset lacks diversity such as including images or video frames at different times of the day, adverse weather conditions and containing larger flocks of seabirds. Increasing the diversity of the FNZ dataset would enable our seabird model to detect seabirds more accurately in various weather and lighting conditions. Furthermore, to solve the single class detection problem, our current FNZ dataset can be expanded by collecting and annotating more images or video frames containing different species of seabirds and a new YOLO model could then be fine-tuned on this dataset for multi-species seabird detection.

## 7. Conclusions

This paper discusses the development of a deep learning model, specifically a modified YOLO model to automatically detect seabirds in unconstrained fish harvesting images as a step towards mitigating seabird bycatch during fishing operations. From the experimental results, the developed fine-tuned YOLOv8 large model achieved the best performance, with a mAP@50 score of 0.9926 and a mAP@50-95 score of 0.9147 on the test set, which show its effectiveness in the real-world marine scenario. This project also developed a basic interactive dashboard to present and visualise the results for our seabird detection model. However, the model is currently restricted to a binary classification problem (i.e. detecting whether an object is a seabird or not) and struggles with detecting the smaller seabirds that are further away from the camera. Future development should include modifying the YOLO architecture to detect smaller seabirds under various weather and lighting conditions and creating a multi-species seabird and bycatch dataset for the YOLO model to detect various species of seabirds and bycatch. The proposed enhancements will make the seabird model more valuable for ecological monitoring such as monitoring seabird interactions with commercial fishing vessels by species. Additionally, increasing the diversity of the FNZ dataset will allow the seabird model to detect seabirds under more challenging and unpredictable environmental conditions such as footages that were captured in different weather conditions. Finally, the seabird model has the potential to be combined with an object tracking algorithm such as ByteTrack or DeepSORT to track and count unique seabirds in video clips, allowing reviewers to gain useful insights into the number of seabirds detected by species.

## Note

1
https://github.com/WongKinYiu/yolov9
